# Advancing genetic testing for neurological disorders in Tanzania: importance, challenges, and strategies for implementation

**DOI:** 10.3389/fnins.2024.1371372

**Published:** 2024-03-13

**Authors:** Mohamed Zahir Alimohamed, Angela Augustine Siima, Mohamed Manji

**Affiliations:** ^1^Department of Biochemistry and Molecular Biology, Muhimbili University of Health and Allied Sciences, Dar es Salaam, Tanzania; ^2^Tanzania Human Genetics Organization, Dar es Salaam, Tanzania; ^3^Department of Genetics, University Medical Center Groningen, Groningen, Netherlands; ^4^Department of Internal Medicine, Muhimbili University of Health and Allied Sciences (MUHAS), Dar es Salaam, Tanzania; ^5^Tanzania Neuroscience Association, Dar es Salaam, Tanzania; ^6^Department of Internal Medicine, Ebrahim Haji Charitable Health Centre, Dar es Salaam, Tanzania

**Keywords:** genetic testing, neurological disorders, capacity building, bioinformatics, Tanzania

## Abstract

This manuscript discusses the critical need for advancing genetic testing capabilities for neurological disorders (NDs) in Tanzania, emphasizing the importance, challenges, and strategies for implementation. Neurological disorders, often caused by a complex interplay of genetic and environmental factors, disproportionately affect low and middle-income countries like Tanzania. Patients with neurogenetic diagnoses in Tanzania face substantial obstacles, including lack of trust in medical professionals, stigmatization, and limited access to proper care. The manuscript underscores the infancy of genetic testing implementation for NDs in Tanzania, citing challenges such as high costs, limited availability, and the absence of routine testing in diagnostic procedures. The paper recommends establishing well-characterized cohorts, conducting a nationwide emergency census survey, and enhancing diagnostic services and healthcare financing. The paper stresses on the importance of collaboration, research, public awareness, and advocacy to address the challenges posed by NDs in Tanzania. The manuscript proposes a comprehensive plan, including training and capacity building in molecular diagnosis and bioinformatics, to establish genetic testing capabilities and reduce the burden of NDs in the country. Overall, the manuscript calls for a collaborative effort involving government support, stakeholder engagement, and international partnerships to advance genetic testing and improve healthcare outcomes for individuals with neurological disorders in Tanzania.

## Introduction

Neurological disorders (NDs) affect the brain, spinal cord, cranial nerves, peripheral nerves, nerve roots, autonomic nervous system, neuromuscular junction, and muscles, and frequently cause significant morbidity and mortality (Di Natale et al., 2021). These can result from structural, biochemical, or electrical abnormalities in the nervous system. These include a wide range of diseases such as Alzheimer’s disease, Parkinson’s disease, Huntington’s, multiple sclerosis, ataxia, epilepsy, muscular dystrophy, etc. Most neurological conditions occur as a complex interplay of genetic and environmental factors. These illnesses have multifactorial causation, and many are hereditary (Butler and Zeman, 2005; Walzl et al., 2019). Generally, in Low and Middle-Income countries (LMIC) like sub-Saharan African countries, the burden of NDs is overwhelming (Lekoubou et al., 2014; Williams et al., 2018). Globally the burden of noncommunicable NDs has been reported to be increasing in recent years (Stovner et al., 2014; GBD 2016 Neurology Collaborators, 2019) causing more than 1% of deaths and 28% of all disabilities.

In Tanzania, patients with neurogenetic diagnoses face significant obstacles in obtaining proper care. They often doubt their symptoms, resorting to traditional or faith healers after unsuccessful visits to medical doctors. Lack of trust in medical professionals is common due to repeated misdiagnoses. Community stigmatization as bewitched or possessed further isolates them. Many remain undiagnosed and untreated, with only a few reaching neurologists for proper care. Experimental treatments, including conventional and herbal therapies, lead to side effects, and mental health issues like depression and anxiety are prevalent. Additionally, patients experience social inequalities in education, employment, and marriage.

Rare Disease Day in Tanzania, organized by The Tanzania Human Genetics Organization (THGO) and the Ali Kimara Rare Diseases Foundation (AKRDF), aims to raise awareness and advocate for those affected by rare diseases (Alimohamed et al., 2021; Kaywanga et al., 2022). The event includes educational programs, workshops, and awareness campaigns for healthcare professionals, policymakers, and the public, emphasizing the importance of early diagnosis, specialized care access, and ongoing research. A dedicated session during Rare Disease Day 2023, organized by THGO, focused on identifying neurogenetic disorders. Participants received training on approaching neurogenetic patients, with examples of conditions such as Essential Tremor, Spinocerebellar Ataxia, Mitochondrial Myopathy, Kearne Sayre Syndrome, Familial Hemiplegic Migraine, Cerebral Autosomal Dominant Arteriopathy with Subcortical Infarcts (CADASIL), and Inborn Errors of Metabolism highlighted.

With the advancement in human genetics over the past few decades, genetic testing for the diagnosis of NDs has emerged as a powerful tool for diagnosing and managing neurological disorders globally (Toft, 2014; Srivastava et al., 2019). The implementation of genetic testing in diagnosis of neurological conditions in Tanzania is still in its infancy. Neurogenetic diagnoses are generally made from a combination of a detailed neurological history and physical exam followed by imaging and other supportive tests. Precise genetic diagnosis based on genetic testing are rarely achieved. Genetic testing is very expensive and out of reach of many patients (Adebayo et al., 2020). Outside of selected published case reports and anecdotal experiences, Tanzanian neurogenetics is largely missing from the global neurogenetic landscape. Published cases include Becker muscular dystrophy (Dekker et al., 2019), Huntington’s disease (Scrimgeour, 1981), Motor neuron disease (Dekker et al., 2018), Parkinson’s disease (Dekker et al., 2020), Carpenter syndrome (Lodhia et al., 2021), Adrenoleukodystrophy (Dekker et al., 2019), Paroxysmal kinesigenic dyskinesia (Dekker et al., 2020), Freeman-Sheldon syndrome (Ali et al., 2017), Cornelia de Lange Syndrome (Mende et al., 2012), Ataxia-Telangiectasia (van Os et al., 2020), and Nodding syndrome (Amaral et al., 2023). It is important to note that all of the above ascertained in Tanzania with molecular confirmation done overseas. Hence the need of being able to develop Human Genetics expertise in all its aspects within the country with the ultimate aim of capacity building but also to have African human genetic material remain in Africa.

Anecdotally, unpublished encounters by clinicians in Tanzania with conditions like Pallister Kilian syndrome; Ataxia Oculomotor Apraxia; SCA; SMA1; SMA2; Brown Vialetto Van Laere etc., have also been noted. Furthermore, there is an ongoing genetic analysis of the national study group of neuro-Xeroderma Pigmentosum patients in KCMC (Moser, 2015).

This manuscript discusses the importance of establishing genetic testing capabilities for neurological disorders in Tanzania, highlighting various aspects that need to be addressed for effective implementation.

### Genetic tests in Tanzania: what has been done

Genetic testing is a rapidly advancing diagnostic tool for inherited neurological illnesses (Salunkhe et al., 2022). It not only provides a full diagnosis but also assesses the risk of inheriting genetic disorders and identifies susceptible family members who may benefit from testing. Next-generation sequencing (NGS) technologies like Pac-Bio, Illumina, and Oxford MinION sequencing enable various approaches, such as targeted panel sequencing, whole-exome sequencing, and whole-genome sequencing, to analyze hundreds of hereditary neurological disorders affecting the brain, muscles, and nerves. Neurological genetic testing plays a crucial role in guiding treatment choices, identifying at-risk relatives, and facilitating participation in potential clinical trials.

Genetic testing in Tanzania is currently at a nascent stage, with limited availability and utilization. While specialist medical facilities and research organizations do offer genetic testing services, they are not widely accessible or commonly practiced. Newborn screening and paternity tests are among the few genetic tests available, but comprehensive testing for various genetic disorders and diseases is not yet a routine practice. A recent case study has shed light on the potential for advancements in genetic testing within Tanzania. This study confirmed the presence of PRKN-related familial Parkinson’s disease using molecular techniques, signaling a promising step forward in the field of genetics in the country (Dekker et al., 2020). In contrast, the broader landscape of genetic testing for neurological disorders and diseases in adults in the northern Tanzania has shown limitations. While various neurological conditions such as stroke, infections, neuropathies, and movement disorders are prevalent, laboratory-based genetic testing is not yet a standard part of diagnostic procedures (Howlett et al., 2022). Overall, there is a pressing need for the expansion of genetic testing capabilities and resources in Tanzania, especially in fields like epilepsy, movement disorders, neuropathies, ataxias and muscular dystrophies, to better address genetic disorders and contribute to improved healthcare outcomes.

### Recommendations and call for action: genetic testing for neurological disorders

#### Establishing cohorts

The establishment of well-characterized cohorts is fundamental to genetic research and testing. In Tanzania, the creation of representative cohorts that encompass diverse ethnicities, geographical regions, and socioeconomic backgrounds is essential. Creating these cohorts for neurological conditions requires careful preparation, ethical considerations, and coordination with key stakeholders. These cohorts would provide valuable insights into the genetic landscape of neurological disorders within the country, facilitating the identification of population-specific genetic variants and contributing to the broader understanding of our genetic architecture.

#### Immediate census on individuals with neurological conditions

Data on neurological disorder incidence and affected individuals are limited in Tanzania, hampering social support, policy formulation, and resource allocation. An urgent effort to conduct a nationwide emergency census survey and baseline research is needed. This data collection effort will provide an overview of the magnitude of the problem due to the absence of genetic tests for neurological conditions. The census will yield information on affected individuals’ characteristics, locations, and the spectrum of neurological diseases. It is recommended to establish a national registry linked to existing health and educational databases. Researchers will gather information on the immediate, short-term, and long-term needs of individuals with neurological disorders. These findings will offer essential insights and recommendations to expedite access to healthcare, education, and services for those with neurological illnesses.

#### Diagnostic services, care, treatment and healthcare financing

Genetic testing provides crucial insights into the risk of acquiring neurological diseases and the likelihood of passing them on to future generations. This information guides informed decisions about family planning, medical treatments, and interventions like preimplantation genetic diagnosis or prenatal testing with genetic counseling. It enables early treatment and appropriate management of neurological disorders (NDs) and facilitates education for family members and caregivers. Creating special identification for individuals with NDs expedites their access to healthcare services and encourages affordable health insurance. Additionally, establishing NDs testing units within relevant laboratories like Muhimbili Genetic Laboratory (MGL) is recommended.

It is imperative to underscore the impact of a scarcity of neurologists, coupled with a lack of essential neurological facilities, such as neurophysiology and neuroimaging, alongside a low rate of healthcare coverage in Tanzania. This dearth poses a significant barrier, as many neurogenetic conditions may not present for analysis due to limited access to specialized medical professionals and diagnostic resources. Moreover, the pressing need for culture-appropriate and -sensitive genetic counseling deserves dedicated attention. In the local Tanzanian context, counseling encounters various challenges, exemplified by limited antenatal ultrasound scans and other check-ups, restricting the comprehensive assessment of potential neurogenetic conditions during crucial stages of development. These challenges underscore the necessity for tailored strategies in genetic counseling that consider the local healthcare landscape, ensuring effective communication and decision-making for families navigating the complexities of neurogenetic disorders.

#### Research, innovation and public private partnership

Comprehensive collaboration involving public and private sectors is essential to address the challenges posed by neurological illnesses. This collaborative effort can drive advancements in genetic testing for NDs, foster research, and enhance patient outcomes. Research holds the potential to provide valuable genetic data, improving the understanding of disease causes and molecular mechanisms, ultimately leading to better diagnosis and treatment for various NDs. Establishing and maintaining a Tanzanian stakeholder network, such as the Tanzania Neuroscience Association network, can serve as a vital platform for public education, advocacy, and awareness, mobilizing support for individuals affected by NDs.

#### Policies, public awareness and advocacy

Raising awareness about the significance of genetic testing for neurological illnesses in Tanzania holds paramount importance, particularly considering the potential to drive policy changes and foster a supportive environment. Acknowledging the vulnerability of mothers in navigating neuro-genetic disorders and the associated stigma and misunderstandings, it is imperative to emphasize their unique challenges. While acknowledging that fathers may also face challenges, the emphasis here is on the vulnerable position of females in these circumstances. Collaboration with healthcare professionals, decision-makers, patient advocacy groups, and the public remains essential. Establishing partnerships with global neuroscience organizations, including universities, NGOs, and businesses, is critical for fostering information sharing and collaborative research initiatives that transcend geographical boundaries.

Recognizing the societal nuances in Tanzanian and Sub-Saharan societies, where the stigma is intensified for neuro-genetic conditions, especially those affecting females, we underscore the need for a nuanced approach to public awareness campaigns and advocacy efforts. Recommendations include setting up genetic counseling and testing centers across Tanzania to provide services for individuals and families affected by NDs.

A dedicated section on culture-appropriate and -sensitive genetic counseling is proposed, delving into everyday examples that illuminate the complexities faced by individuals and families in the local Tanzanian context. The section aims to shed light the societal taboo surrounding discussions on pregnancy termination, consanguinity, and birth control. Addressing these challenges within the cultural and legal frameworks is crucial, and we advocate for the inclusion of these considerations in genetic counseling practices.

Moreover, it is recommended that individuals with neuro-genetic disorders be encouraged to apply for government and health insurance packages. To alleviate financial burdens, public healthcare facilities should explore the possibility of waiving medical expenses for prescribed drugs and services. Priority access to healthcare, especially for vulnerable groups like the elderly and expectant women affected by neuro-genetic disorders, should be a cornerstone of healthcare policy at all levels.

These recommendations are integral to the broader goal of incorporating a comprehensive, culturally sensitive, and patient-centered approach into policies, public awareness campaigns, and advocacy efforts. By aligning these initiatives with the socio-cultural realities of Tanzania, we aim to pave the way for meaningful change and improved healthcare outcomes for individuals and families affected by neuro-genetic disorders. Sharing knowledge, skills, and infrastructure between policymakers and researchers can expedite research processes, aligning research objectives with market demands and promoting economically viable solutions.

#### Training, capacity building in molecular diagnosis and bioinformatics

The integration of molecular biology and bioinformatics has revolutionized biological research, enabling the analysis of vast amounts of genetic and protein data. Collaboration and data sharing have driven progress in genetics and related fields. To advance genetic testing for NDs, a comprehensive plan for training individuals in this domain is essential. Developing local bioinformatics expertise is crucial for effective data analysis, variant interpretation, and clinical integration. Furthermore, creating molecular diagnostic tools tailored to the Tanzanian population’s unique needs can aid in identifying genetic variations responsible for NDs. Establishing relevant training programs and infrastructure for genetic testing is essential to enhance local expertise, capacity, and understanding of NDs.

## Discussion

This manuscript highlights the underrepresentation of the Tanzanian population in the global genomic database for NDs due to limited information and lacking diagnostic tools, including genetic tests. To address this issue and improve the understanding and treatment of NDs in Tanzania, collaboration between national and international researchers is essential. It presents the first comprehensive policy recommendation for developing genetic testing for ND diagnosis in Tanzania, a critical step in advocating for the rights and well-being of Tanzanians affected by NDs. We propose a Call for Action as the initial step to involve the Tanzanian government and stakeholders in addressing these challenges and improving research and clinical diagnosis to mitigate the burden of NDs in Tanzania. Despite the efforts of neurologists and researchers, there is limited public understanding of neurological illnesses in Tanzania, leading to stigma and discrimination against those affected. Misdiagnosis or inconclusive diagnoses of NDs contribute to this issue. Implementing genetic testing can offer clear and definitive diagnoses for specific NDs, aiding healthcare providers and families in making informed decisions regarding treatment, therapy, disease management, and lifestyle adjustments.

Several studies conducted in Tanzania, including those in Dar es Salaam, Moshi, Kilimanjaro, and the University of Dar es Salaam, have identified over twenty neurological disorders (NDs) in urban populations, encompassing motor, muscular, and cognitive NDs (Laizer et al., 2018; Adebayo et al., 2020; Siima et al., 2020; Stephano, 2021; Howlett et al., 2022). However, none of these studies employed genetic tests for diagnosis. A pediatric professor and student at Muhimbili University of Health and Allied Sciences (MUHAS) have reported data on Autism Spectrum Disorder (ASD), a common ND affecting child neurodevelopment (Ruparelia, 2021). We call upon the Tanzanian government, particularly the Ministry of Health, to prioritize NDs in research and provide increased support to research institutions. This aligns with the global action plan (WHO, 2022) outlined by the World Health Organization (WHO), emphasizing the need for resource mobilization strategies and investments in areas such as early interventions, new diagnostic technologies, and specialist training, especially in low- and middle-income countries like Tanzania.

The use of molecular techniques for the clinical diagnosis of diseases is an evolving science that we should use to our advantage to reduce the cases of complicated and inconclusive diagnosis of genetic conditions including NDs. Therefore, additional work is still required, and a strict plan will be needed to address major issue of genetic testing and equal access to testing, representation of all populations, and harnessing and adapting to technological changes in the field.

## Conclusion

To enhance ND diagnosis and care in Tanzania, establishing genetic testing capabilities is vital. This entails addressing underrepresentation in global genomic databases, implementing training and research programs, enhancing bioinformatics and molecular diagnostics, securing funding, gaining government support, involving stakeholders, and forming international partnerships. These efforts will improve healthcare and reduce the ND burden. By working together to overcome challenges, we can advance healthcare outcomes, provide personalized care, and deepen our understanding of NDs, ensuring equitable access to genetic testing and healthcare for all Tanzanians.

## Data availability statement

The original contributions presented in the study are included in the article/supplementary material, further inquiries can be directed to the corresponding author.

## Author contributions

MA: Conceptualization, Writing – original draft, Writing – review & editing. AS: Writing – original draft, Writing – review & editing. MM: Writing – original draft, Writing – review & editing.

## References

[ref1] Adebayo P. B. Aziz O. M. Mwakabatika R. E. Makakala M. C. Mazoko M. C. Adamjee S. M.. (2020). Out-patient neurological disorders in Tanzania: experience from a private institution in Dar Es Salaam. eNeurologicalSci 20:100262. doi: 10.1016/j.ensci.2020.100262, PMID: 32802973 PMC7417890

[ref2] Ali A. M. Mbwasi R. M. Kinabo G. Kamsteeg E. J. Hamel B. C. Dekker M. C. J. (2017). Freeman-Sheldon syndrome: first molecularly confirmed case from sub-Saharan Africa. Case Rep. Genet. 2017:9327169. doi: 10.1155/2017/9327169, PMID: 28584669 PMC5443993

[ref3] Alimohamed M. Z. Mwakilili A. D. Mbwanji K. Manji Z. K. Kaywanga F. Mwaikono K. S.. (2021). Inauguration of the Tanzania Society of Human Genetics: biomedical research in Tanzania with emphasis on human genetics and genomics. Am. J. Trop. Med. Hyg. 104, 474–477. doi: 10.4269/ajtmh.20-0861, PMID: 33350369 PMC7866332

[ref4] Amaral L. J. Bhwana D. Mhina A. D. Mmbando B. P. Colebunders R. (2023). Nodding syndrome, a case-control study in Mahenge, Tanzania: Onchocerca volvulus and not Mansonella perstans as a risk factor. PLoS Negl. Trop. Dis. 17:e0011434. doi: 10.1371/journal.pntd.0011434, PMID: 37339148 PMC10313037

[ref5] Butler C. Zeman A. Z. (2005). Neurological syndromes which can be mistaken for psychiatric conditions. J. Neurol. Neurosurg. Psychiatry 76, i31–i38. doi: 10.1136/jnnp.2004.060459, PMID: 15718219 PMC1765684

[ref6] Dekker M. C. J. Chengo R. Kumburu H. H. Kamsteeg E. J. Hamel B. C. (2020). Paroxysmal Kinesigenic dyskinesia: first molecularly confirmed case from Africa. Tremor. Other Hyperkinet. Mov. (N. Y.) 10, 1–12. doi: 10.7916/tohm.v0.742 32002278 PMC6982423

[ref7] Dekker M. C. J. Sadiq A. M. Mc Larty R. Mbwasi R. M. Willemsen M. A. A. P. Waterham H. R.. (2019). A Tanzanian boy with molecularly confirmed X-linked Adrenoleukodystrophy. Case Rep. Genet. 2019:6148425. doi: 10.1155/2019/6148425, PMID: 32089906 PMC7011349

[ref8] Dekker M. C. J. Suleiman J. M. Bhwana D. Howlett W. P. Rashid S. M. van Minkelen R.. (2020). PRKN-related familial Parkinson's disease: first molecular confirmation from East Africa. Parkinsonism Relat. Disord. 73, 14–15. doi: 10.1016/j.parkreldis.2020.02.014, PMID: 32193156

[ref9] Dekker M. C. J. Tieleman A. A. Igogo O. J. van Duyvenvoorde H. A. Howlett W. P. Hamel B. C. (2019). First familial Becker muscular dystrophy in Tanzania: clinical and genetic features. Neuromuscul. Disord. 29, 317–320. doi: 10.1016/j.nmd.2019.01.006, PMID: 30926200

[ref10] Dekker M. C. J. Urasa S. J. Aerts M. B. Howlett W. P. (2018). Motor neuron disease in sub-Saharan Africa: case series from a Tanzanian referral hospital. J. Neurol. Neurosurg. Psychiatry 89, 1349–1350. doi: 10.1136/jnnp-2017-317858, PMID: 29549191

[ref11] Di Natale M. R. Stebbing M. J. Furness J. B. (2021). Autonomic neuromuscular junctions. Auton. Neurosci. 234:102816. doi: 10.1016/j.autneu.2021.102816 33991756

[ref12] GBD 2016 Neurology Collaborators (2019). Global, regional, and national burden of neurological disorders, 1990-2016: a systematic analysis for the global burden of disease study 2016. Lancet. Neurol. 18, 459–480. doi: 10.1016/S1474-4422(18)30499-X, PMID: 30879893 PMC6459001

[ref19] Howlett W. P. J Urasa S. P Maro V. W Walker R. G Kilonzo K. J Howlett P.. (2022). Neurological disorders in northern Tanzania: a 6-year prospective hospital-based case series. Afr. Health Sci. 22, 269–284. doi: 10.4314/ahs.v22i1.34, PMID: 36032440 PMC9382522

[ref13] Kaywanga F. Alimohamed M. Z. David A. B. Maeda D. Mbarak S. Mavura T.. (2022). Rare diseases in Tanzania: a National Call for action to address policy and urgent needs of individuals with rare diseases. Orphanet J. Rare Dis. 17:343. doi: 10.1186/s13023-022-02498-0, PMID: 36064429 PMC9446714

[ref14] Laizer S. Kilonzo K. Urasa S. Maro V. Walker R. Howlett W. (2018). Neurological disorders in a consultant hospital in northern Tanzania. A cohort study. eNeurologicalSci 14, 101–105. doi: 10.1016/j.ensci.2018.11.013, PMID: 30828651 PMC6382946

[ref15] Lekoubou A. Echouffo-Tcheugui J. B. Kengne A. P. (2014). Epidemiology of neurodegenerative diseases in sub-Saharan Africa: a systematic review. BMC Public Health 14:653. doi: 10.1186/1471-2458-14-653, PMID: 24969686 PMC4094534

[ref16] Lodhia J. Rego-Garcia I. Koipapi S. Sadiq A. Msuya D. Spaendonk R. V.. (2021). Carpenter syndrome in a patient from Tanzania. Am. J. Med. Genet. A 185, 986–989. doi: 10.1002/ajmg.a.62015, PMID: 33368989

[ref17] Mende R. H. Drake D. P. Olomi R. M. Hamel B. C. (2012). Cornelia de Lange syndrome: a newborn with imperforate anus and a NIPBL mutation. Case Rep. Genet. 2012:247683. doi: 10.1155/2012/247683, PMID: 23304577 PMC3529426

[ref18] Moser R. (2015). Regional dermatology training center, Moshi, Tansania--das Herz der Dermatologie in Ostafrika [regional dermatology training center Moshi, Tanzania—the heart of dermatology in East Africa]. J. Dtsch. Dermatol. Ges. 13, 612–613. doi: 10.1111/ddg.12713 26018386

[ref20] Ruparelia K. (2021). Autism Spectrum disorders in Tanzania: awareness, diagnosis, risk factors and Endophenotypes. PhD thesis Dar es salaam, Tanzania: The Open University.

[ref21] Salunkhe M. Agarwal A. Faruq M. Srivastava A. K. (2022). Genetic testing in neurology: what every neurologist must know. Ann. Indian Acad. Neurol. 25, 350–353. doi: 10.4103/aian.aian_855_21, PMID: 35936600 PMC9350807

[ref22] Scrimgeour E. M. (1981). Huntington's disease in Tanzania. J. Med. Genet. 18, 200–203. doi: 10.1136/jmg.18.3.200, PMID: 6453998 PMC1048705

[ref23] Siima A. A. Stephano F. Munissi J. J. E. Nyandoro S. S. (2020). Ameliorative effects of flavonoids and polyketides on the rotenone induced Drosophila model of Parkinson's disease. Neurotoxicology 81, 209–215. doi: 10.1016/j.neuro.2020.09.004, PMID: 32937168

[ref24] Srivastava S. Love-Nichols J. A. Dies K. A. Ledbetter D. H. Martin C. L. Chung W. K.. (2019). Meta-analysis and multidisciplinary consensus statement: exome sequencing is a first-tier clinical diagnostic test for individuals with neurodevelopmental disorders. Genet. Med. 21, 2413–2421. doi: 10.1038/s41436-019-0554-6, PMID: 31182824 PMC6831729

[ref25] Stephano F. (2021). Baphia macrocalyx leaf extract attenuates rotenone-induced toxicity in *Drosophila melanogaster* model of Parkinson’s disease. J. Educ. Human.(1 SE, 125–136. Available at: http://ojs3.bongotech.info/index.php/jehs/article/view/77

[ref26] Stovner L. J. Hoff J. M. Svalheim S. Gilhus N. E. (2014). Neurological disorders in the global burden of disease 2010 study. Acta Neurol. Scand. Suppl. 129, 1–6. doi: 10.1111/ane.12229 24588499

[ref27] Toft M. (2014). Advances in genetic diagnosis of neurological disorders. Acta Neurol. Scand. Suppl. 129, 20–25. doi: 10.1111/ane.12232 24588502

[ref28] van Os N. J. van Aerde K. J. van Gaalen J. Merkus P. J. Silveira-Moriyama L. Tajudin T. A.. (2020). Diagnosis and Management of Ataxia-Telangiectasia in resource-limited settings. J. Int. Child Neurol. Ass. 1, 1–12. doi: 10.17724/jicna.2020.181

[ref29] Walzl D. Carson A. J. Stone J. (2019). The misdiagnosis of functional disorders as other neurological conditions. J. Neurol. 266, 2018–2026. doi: 10.1007/s00415-019-09356-3, PMID: 31115678 PMC6647145

[ref30] WHO (2022). Draft Intersectoral global action plan on epilepsy and other neurological disorders 2022–2031 World Health Organisation, 1–28 Available at: https://cdn.who.int/media/docs/default-source/brain-health/first-draft-action-plan-on-epilepsy-and-other-neurological-disorders_180621.pdf. 10.1227/neu.0000000000001976 35383690

[ref31] Williams U. Bandmann O. Walker R. (2018). Parkinson's disease in sub-Saharan Africa: a review of epidemiology, genetics and access to care. J. Mov. Disord. 11, 53–64. doi: 10.14802/jmd.17028, PMID: 29860783 PMC5990907

